# The Organization and Connections of Second Somatosensory Cortex in the Agouti

**DOI:** 10.3389/fnana.2018.00118

**Published:** 2019-01-14

**Authors:** Lucidia F. Santiago, Marco Aurelio M. Freire, Cristovam W. Picanço-Diniz, João G. Franca, Antonio Pereira

**Affiliations:** ^1^Laboratory of Investigations in Neurodegeneration and Infection, Institute of Biological Sciences, Federal University of Pará, Belém, Brazil; ^2^Laboratory of Experimental Neuroprotection and Neuroregeneration, Institute of Biological Sciences, Federal University of Pará, Belém, Brazil; ^3^Institute of Biophysics Carlos Chagas Filho, Federal University of Rio de Janeiro, Rio de Janeiro, Brazil; ^4^Institute of Technology, Federal University of Pará, Belém, Brazil

**Keywords:** S2, somatotopic map, cortical connections, agouti, rodent

## Abstract

In order to understand how the mammalian sensory cortex has been structured during evolution, it is necessary to compare data from different species across distinct mammalian lineages. Here, we investigated the organization of the secondary somatosensory area (S2) in the agouti (*Dasyprocta aguti*), a medium-sized Amazonian rodent, using microelectrode mapping techniques and neurotracer injections. The topographic map obtained from multiunit electrophysiological recordings were correlated with both cytochrome oxidase (CO) histochemistry and with patterns of corticocortical connections in tangential sections. The electrophysiological mapping of the lateral strip of parietal cortex adjacent to the primary somatosensory area (S1) revealed that S2 displays a mirror-reversed topographical representation of S1, but with a smaller cortical magnification factor. The caudal border of S2 is surrounded by sensory fields which also respond to auditory stimulation. BDA injections into the forelimb representation of S2 revealed a dense homotopic ipsilateral projection to S1, supplemented by a less dense projection to the caudolateral cortex located near the rhinal sulcus (parietal rhinal area) and to a frontal region probably associated with the motor cortex. Our findings were similar to those described in other mammalian species, reinforcing the existence of a common plan of organization for S2 in the mammalian parietal cortex.

## Introduction

The organization of the mammalian somatosensory cortex has been the subject of scrutiny since the pioneer studies of Gerard et al. (1933) and Marshall et al. (1937). In these works, the authors identified an area responsive to tactile stimuli located in the parietal lobe of both monkeys and cats (Gerard et al., 1933; Marshall et al., 1937). The experimental evidence for a secondary somatosensory area (S2) was first provided by Adrian (1940), who described an additional cortical representation of the cat’s feet next to the previously defined “first” somatosensory area (Adrian, 1940). Later on, with further development of microelectrode recording techniques and the rise of modern anatomical tract-tracing methods, the definition of both the organization and the limits of cortical areas became more reliable, enabling the identification of other somatosensory areas that had not previously been described, such as the parietal ventral (PV) and parietal rhinal (PR) areas (Krubitzer et al., 1986; Fabri and Burton, 1991).

The existence of multiple somatosensory fields in the cortex is a feature shared by all mammals (Kaas, 2011). Since monotremes, such as the platypus and the echidna, whose lineage emerged very early from the mammalian evolutionary branch (Divac, 1995), possess three separate somatosensory fields, S1, S2/PV, and a rostral field (R) (Krubitzer, 1998), it is argued that the multiplicity of tactile representations is a characteristic of the mammalian therapsid ancestors as well (Krubitzer and Kahn, 2003). The number of sensory fields seems to be closely associated with increments in both the complexity and scope of mammalian behavior (Kaas, 1989). According to this view, each sensory area is responsible for the extraction of different information from the environment while providing a connection with motor circuits controlling purposeful movements (Metzner and Juranek, 1997). For instance, in humans, whose behavior displays a strong reliance on vision, more than 20% of the entire cortex is devoted to visual processing distributed in about forty visual areas (Kaas, 1989; Wandell et al., 2007). However, the same is not true for the somatosensory modality, with most studies acknowledging the existence of only S1 and S2 and their intrinsic cytoarchitectonic subdivisions in humans (Eickhoff et al., 2006a,b).

Throughout evolution, the emergence of sensory fields in the cortex is shaped by the interplay between functional (Patel et al., 2014) and developmental constraints (Huntenburg et al., 2017). To get a clear picture of how these constraints interact, it is necessary to perform studies investigating the correlation of brain features with ecological and behavioral adaptive variables in species with different lifestyles, since a comparative approach is essential for the understanding of brain organization and function (Moss, 2018). Rodents comprise the largest and more diversified order among mammals, being represented by more than two thousand species grouped into 34 families (Wilson and Reeder, 2005) displaying the broadest ecological diversity and are a valuable target for such studies. Rodents are particularly diversified in South America, which remained isolated from other continents during the Cenozoic era and is now home to some of the biggest rodents on Earth, such as the genera Dasyprocta, Cuniculus, and Hydrochoerus (Nowak and Walker, 1999). South American rodents occupy a variety of habitats, ranging from the fossorial tuco-tuco (*Ctenomys*), the arboreal spiny rats (*Echimyidae*), the terrestrial agouti (*Dasyprocta*), and the semi-aquatic capybara (*Hydrochoerus*).

**FIGURE 1:**
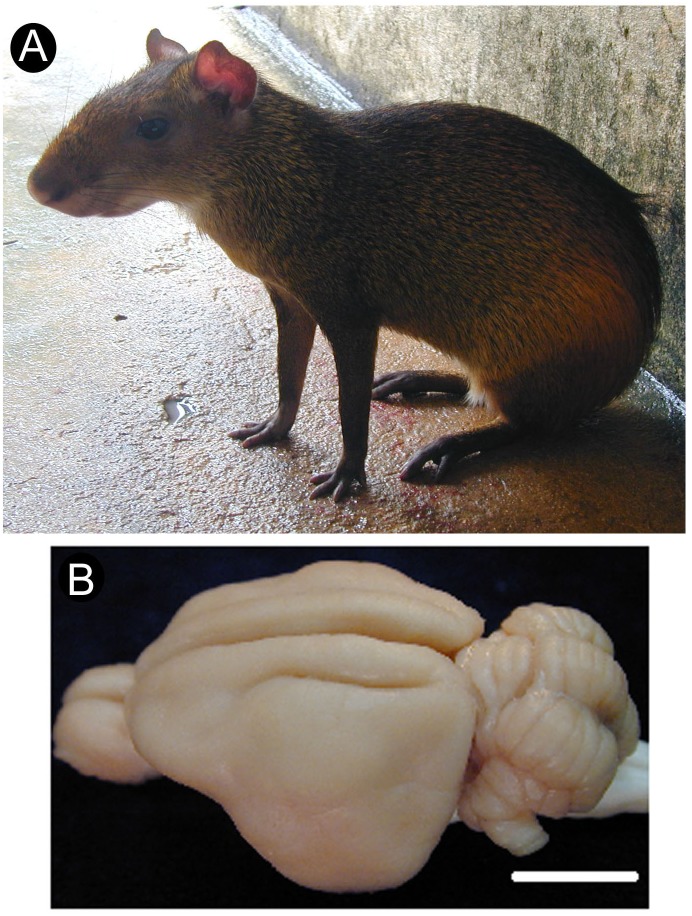
Agouti in its typical feeding posture, sitting on its hindlimbs **(A)**. Dorsolateral view of the agouti’s brain with its lisencephalic aspect **(B)**. Scale bar: 1 cm.

Despite the great variety of rodent species, however, our understanding of the organization and function of the rodent cortex is based mostly on results from two murine species: the house mouse (*Mus musculus*) and the laboratory rat (*Rattus norvegicus*) (Manger et al., 2008). This lack of diversity of experimental models hinders the goal of understanding the role of evolutionary constraints on cortical areal organization (Krubitzer, 1995; Catania and Kaas, 1997, 2001; Catania et al., 2000a; Catania and Remple, 2002; Patzke et al., 2014; Dooley et al., 2015; Jacobs et al., 2015). Along the years, our group has provided information on the organization of the central nervous system of a medium-sized (2.5–4.0 kg) South American rodent, the agouti (*Dasyprocta aguti*) (Figure 1A), including the visual (Picanço-Diniz et al., 1989; Elston et al., 2006; Freire et al., 2010; Rocha et al., 2012) and somatosensory cortices (Rocha et al., 2007; Santiago et al., 2010), as well as the spinal cord (Freire et al., 2008). The agouti possesses a relatively large brain compared to other rodents (Santiago et al., 2007) (see Figure 1B) and departs from many ways from common murine laboratory models in terms of body size, habitat, and behavior. Agoutis’ behavior is characterized by being primarily diurnal and by its frugivorous nature, including the consumption of seeds and fruit pulp the animals had previously buried in caches or found on the forest floor (Henry, 1999). Interestingly, large portions of neural tissue both in the somatosensory thalamus (Campos et al., 1972) and S1 (Dias et al., 2014) receive projections from the forepaws and oral structures (lips, incisors) which are extensively used in eating behavior.

There are relatively few studies in rodents evaluating intracortical connections originating from S2 (Carvell and Simons, 1987; Koralek et al., 1990; Alloway et al., 2000). In the present investigation we try to fill this gap by providing information about the organization and hodological pattern of the agouti’s S2 using a combination of multiunit electrophysiological recordings, tract-tracing with biotinylated dextran amine (BDA), and cytochrome oxidase (CO) histochemistry.

## Materials and Methods

### Animals and Ethics Statement

We used six male adult agoutis (*D. aguti*) (2600 ± 175 g), specimens donated by the Emilio Goeldi Zoo-Botanic Museum, under license from the Brazilian Institute of the Environment and Renewable Natural Resources (IBAMA) (ID 207419-0030/2003). All experimental procedures were approved by our institution’s animal care and use committee (CEPAE-UFPA BIO001-10) and all efforts were made to avoid animal distress and to reduce the number of specimens used.

### Electrophysiological Recordings and Neurotracer Injection

Animals were initially anesthetized with a mixture of ketamine hydrochloride (46 mg/kg) and xylazine hydrochloride (4 mg/kg) (i.m.). Throughout the course of the experiment, supplementary doses of the anesthetic mixture were administrated with a minimum interval of 1 h or whenever necessary. The anesthesia state was evaluated by monitoring the animal’s respiration rate, cardiac rhythm, and both eye-blink and foot-pinch reflexes. After the animals were deeply anesthetized, they were placed in a stereotaxic head holder (Narishige Scientific Instruments, Tokyo, Japan) and a wide craniotomy was performed to expose the underlying somatosensory cortex of the left hemisphere. The dura mater was reflected, and the exposed cortical surface was protected with agar.

Recordings were made with low-impedance tungsten microelectrodes (0.8–1.2 MΩ; FHC Inc., Bowdoinham, ME, United States). Electrode penetrations were made perpendicular to the cortical surface and each recording site was marked on an enlarged photograph of the exposed cortex. Blood vessels were used as landmarks to locate the position of electrode penetrations along the cortical surface. The neuronal signals were amplified, filtered, and sent to both an oscilloscope (1476A, BK Precision Inc., Yorba Linda, CA, United States) and a loudspeaker for the monitoring of multiunit receptive field (RF) responses. Somatosensory stimulation on the contralateral body surface consisted of light touches or pressure on the skin, deflection of hairs with a paintbrush, and gentle joint movements. We performed multiple sensory stimulations per recording site. Receptive fields were marked in drawings of the contralateral surface of the animal. In electrode penetrations aimed at regions posterior and lateral to S2, visual and auditory responses were also tested using light flashes and metallic clicks or taps, respectively.

Following the electrophysiological recordings, 0.4 μl of lysine-fixable biotinylated dextran amine (BDA, molecular weight 10,000; Molecular Probes Inc., Eugene, OR, United States) was injected by pressure into the electrophysiologically defined representation of the forelimb using a micropipette attached to a 1 μl Hamilton microsyringe (Hamilton Company, Reno, NV, United States), which was introduced about 600 μm deep into the cortical mantle. After the injection of the neurotracer, the pipette was left stationary inside the cortex for 5 min before being slowly withdrawn. The animals were then recovered from anesthesia and after a survival time of 15–30 days they were submitted to a new recording session in which four to six electrolytic lesions were made to allow reconstruction of electrode penetrations.

### Perfusion and Tissue Processing

After the final electrophysiological recording, the animals were deeply anesthetized with urethane (1.6 g/kg) and perfused transcardially with 0.9% saline followed by 4% paraformaldehyde in 0.1 M phosphate buffer (PB) (pH 7.4). The cortical hemispheres were separated from other brain structures, flattened between two glass slides, and left immersed in 0.1 M PB overnight. Both hemispheres were cut into 100 μm thick sections with a Vibratome (Pelco International, Series 1000, Ted Pella Inc., Redding CA, United States). To reveal the BDA labeling, sections were first incubated overnight in a solution of the avidin-biotin complex (Vectastain ABC kit, Vector Laboratories, Burlingame, CA, United States; 1:200) and subsequently processed following the DAB/nickel method (Shu et al., 1988). Alternate sections were incubated in a CO solution containing 0.05% diaminobenzidine (DAB), 0.03% cytochrome c and 0.02% catalase in 0.1 M PB (Wong-Riley, 1979). Finally, sections were mounted in gelatin-coated glass slides, left to air-dry overnight, dehydrated, cleared in xylene, and coverslipped with Entellan (Merck, Darmstadt, Germany).

### Tissue Reconstruction

Camera Lucida drawings of the histological sections were made at low magnification with a Zeiss Stemi SV 11 optical microscope. The drawings included the following elements: section’s outline, presence of BDA and/or CO labeling, electrolytic lesions, and major anatomical features (e.g., sulci). The drawings of all sections from a given hemisphere were superimposed digitally with the Canvas software (ACD Systems Inc., Fort Lauderdale, FL, United States) using electrolytic lesions and blood vessels as landmarks. This procedure resulted in a combined 2-D reconstruction that incorporated both the somatotopic map obtained with the electrophysiological recordings and the information relative to both BDA and CO labeling. Photomicrographs were obtained with a Nikon AFX-DX Optiphot microscope (Tokyo, Japan). The contrast and/or brightness of pictures were adjusted with the Photoshop CS6 software (Adobe Systems Inc., San José, CA, United States). The areal extension of the representation of body parts in S2 was measured with the ImageJ software^1^, based in the electrophysiological maps (mean values from five electrophysiological maps), and expressed in percentiles, with the whole S2 representation corresponding to 100%. Numerical values are expressed as mean ± standard error of the mean (SEM). Statistical analysis was made using GraphPad Prism 5.0 (GraphPad Software Inc., La Jolla, CA, United States).

## Results

### Somatotopic Organization of S2

**FIGURE 2:**
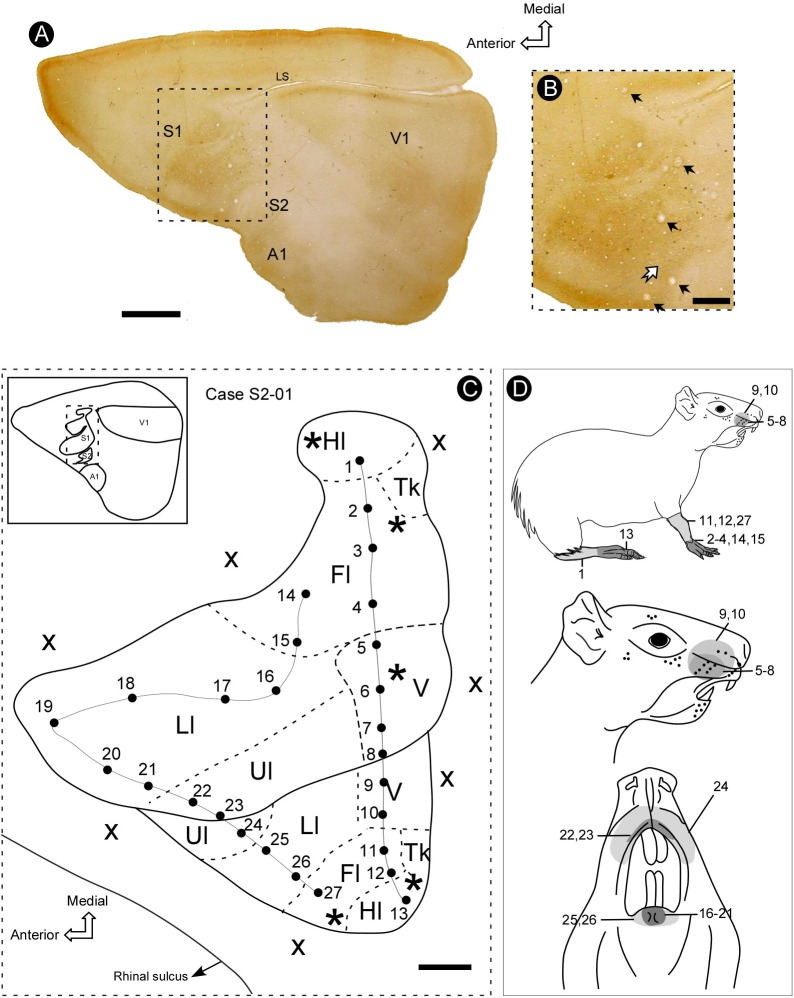
Topographic organization of primary (S1) and secondary (S2) somatosensory areas of the agouti. **(A)** Section of the flattened cortex, processed for cytochrome oxidase (CO) histochemistry, revealing the location of primary somatosensory (S1), auditory (A1), and visual (V1) areas, as well as the secondary somatosensory cortex (S2). **(B)** Micrograph amplification showing S1 and S2 (medial portion) (dotted rectangle in **A**). Electrolytic lesions are indicated by black arrows. The limit between S1 and S2 is defined as a narrow strip of less reactive tissue (white arrow). **(C)** Topographic map of S1 and S2 reconstructed from recording sites. The limits of distinct body representations are delimited by dashed lines. Continuous lines indicate the reversal of receptive field sequences across S1 and S2 (penetrations 1–13 and 14–27). **(D)** Drawing of the agouti’s body indicating the location of receptive fields illustrated in panel **C**. Grayscale codes indicate the difference in the size of receptive fields in both areas. Scale bars: **A**: 5 mm; **B,C**: 2 mm. Hl, hindlimb; Fl, forelimb; V, vibrissae; Ll, lower lip; Ul, upper lip; Tk, trunk; asterisk, electrolytic lesion; X, no response; LS, lateral sulcus.

**FIGURE 3:**
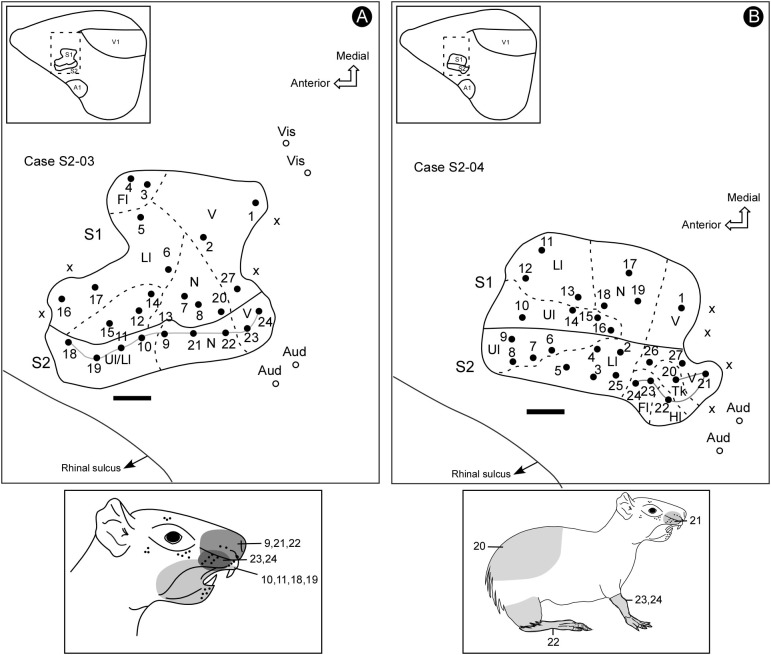
Electrophysiological maps showing the organization of S2 and its relative location in the parietal cortex (inserts). S2 lies in a more lateral and posterior location in the parietal cortex, adjacent to the S1’s face representation, containing a complete representation of the contralateral sensory periphery, which is smaller and has a mirror-symmetrical orientation compared to S1 **(A,B)**. Receptive fields for associated electrode penetrations in cases S2-03 and S2-04 (continuous lines in maps in **A,B**). The shaded areas in the schematic drawings indicate the receptive fields for corresponding electrode penetrations in the electrophysiological maps. Scale bars: 1 mm. Fl, forelimb; Hl, hindlimb; Ll, lower lip; N, nose; Tk, trunk; Ul, upper lip; V, vibrissae; X, no response.

We made a total of one hundred and forty-seven (147) microelectrode penetrations. The electrophysiological recordings revealed a complete representation of the contralateral body surface in S2, located just caudal and lateral to S1 (Figure 2). In all individual cases, the topographical representation of the contralateral body surface in S2 appeared as a mirror-reversed image of S1. Located in a lateral strip of the parietal cortex, S2 is arranged with a rostral to caudal orientation with the representation of both limbs directed toward the lateral border of the cortex (see Figure 3).

The border between S1 and S2 was located along the representation of the head in both fields. The criteria employed to estimate the areal limits included (i) the duplication of the representation of specific contralateral body parts and (ii) the reversion in the progression of receptive fields (RFs) along a continuous row of recording penetrations (Figure 2). In case S2-01, for instance, there is a topographic progression from the forelimb to lower and upper lip representations and back to lower lip and forelimb representations in a more lateral and caudal region, which corresponds to S2 (Figure 2). A clear reversion of the progression of RFs is noticed in electrode penetrations 20–26 (Figure 2C). Another RF sequence reversion can be seen in a sequence of penetrations along hindlimb and forelimb representations in S1 and then back to the hindlimb representation in S2 (Figure 2C).

The topography of the face representation in S2 has an anteroposterior disposition similar to S1, although with a mirror-reversed orientation and being smaller in size. In case S2-03 (Figure 3A), it is possible to notice that the more anterior and lateral recording sites have RFs with representations of both the upper and lower lips. In addition, the more posterior sites respond to both nose and vibrissae stimulation. The representation of the forelimb was situated lateral to the representation of the face and anterior to both the trunk and hindlimb representations (Figure 3B, case S2-04). The representation of the trunk was located close to the representation of the face at the caudal border of S2. The representation of the hindlimb was located at more caudal and lateral segments of S2 (Figure 3B).

**FIGURE 4:**
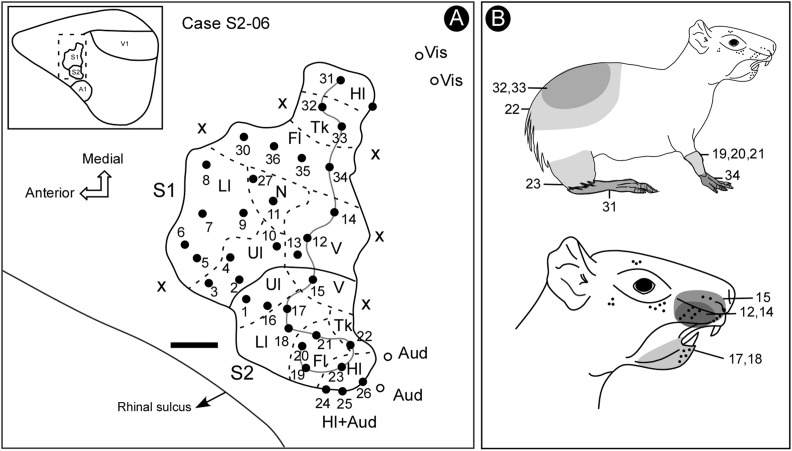
Electrophysiological map (case S2-06) showing the location and organization of S2. Some bimodal responses were identified in a lateral region of S2, across the border with the auditory cortex. In more medial regions, beyond S1, it was possible to identify some visual responses. Notice the reversion in the progression of receptive field locations from S1 to S2 (continuous line) **(A)**. The shaded areas in the schematic drawing indicate the receptive fields associated with corresponding electrode penetrations in the electrophysiological map, indicated by a continuous line **(B)**. Scale bar: 1 mm. Fl, forelimb; Hl, hindlimb; Ll, lower lip; N, nose; Tk, trunk; Ul, upper lip; V, vibrissae; X, no response.

Some electrode penetrations, like those in cases S2-03 and S2-04 (Figures 3, 4), were exclusively responsive to auditory stimulation. These recording sites neighbored the region associated with the trunk and hindlimb representation in S2 (Figure 3B), where bimodal responses were also sometimes obtained (somatosensory plus auditory) (Figure 4). The presence of auditory responses in some electrode recordings was probably associated with the transition zone between S2 and the primary auditory cortex.

Both the anterior and the posterior boundaries of S2 were defined by the absence of somatosensory responses, whilst the lateral limit was defined by the presence of auditory activity at sites located close to the rhinal sulcus (Figure 4).

### Size of Individual Cortical Representations

**FIGURE 5:**
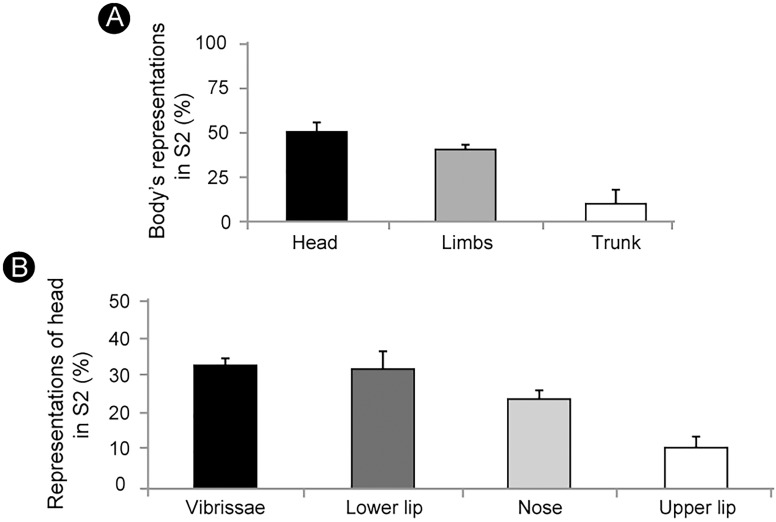
The cortical area occupied by the representation of different body parts in S2. **(A)** About half of S2 is devoted to the representation of the head, followed by the representation of limbs (forelimb: 23%; hindlimb: 17%) and trunk (10%). **(B)** Vibrissae (33%) and lower lip (32%) represent two thirds of the head’s representation, followed by the nose (24%) and upper lip (11%) (mean ± SEM).

Based on measurements of the somatotopic map obtained with the microelectrode recordings, we were able to estimate the surface area occupied by the representation of specific body parts in S2. Thus, half of S2 is devoted to the representation of the face, followed by the representations of the forelimb (23%), hindlimb (17%) and trunk (10%) (Figure 5A) (mean values from five electrophysiological maps).

Within the representation of the head the larger portion is devoted to the vibrissae (33%) and the lower lip (32%), while the nose and the upper lip occupy 24% and 11% of the area, respectively (Figure 5B) (mean values from five electrophysiological maps).

### Cytochrome Oxidase Reactivity

**FIGURE 6:**
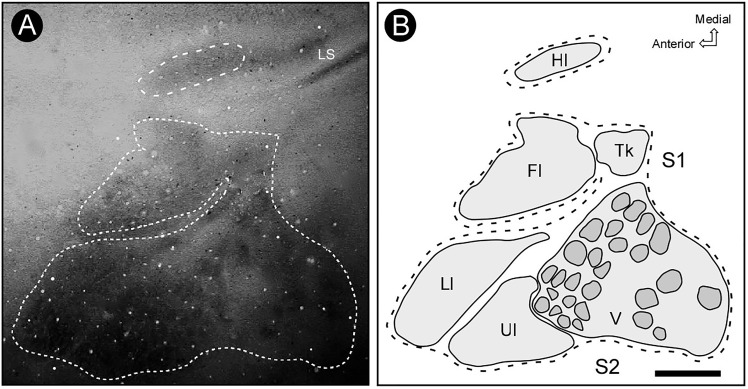
Pattern of cytochrome oxidase (CO) reactivity across S1 area. **(A)** The CO pattern of distribution delimits precisely the distinct representations of the contralateral surface, which are congruent with the electrophysiologically defined borders – lips, limbs, trunk and also modules corresponding to individual vibrissae, in homology to the barrel field of small rodents (mouse and rat). **(B)** Reconstruction of histochemical limits of distinct regions in S1, including cortical modules in the representation of the face. Scale bar: 2 mm. Fl, forelimb; Hl, hindlimb; Ll, lower lip; Tk, trunk; Ul, upper lip; V, vibrissae; LS, lateral sulcus.

In the brain sections reacted for CO (see Figure 2), the histochemical pattern found in S2 differed from that in S1. While the former appeared as a region of moderate staining intensity, the later was more intensely reacted. With the help of CO histochemistry it was possible to discern the border between the two areas, which was characterized by a narrow strip of a strongly reactive cortex (Figure 2). It was possible in S1 to identify labeling patterns associated with distinct representations of the body, such as lips, limbs, trunk and also modules corresponding to individual vibrissae, in homology to the barrel field of small rodents (mouse and rat) (Figure 6); such subdivisions were not identified in S2 (Figure 2). Putative auditory and visual areas, identified by comparison with previously published work (Picanço-Diniz et al., 1989; Elston et al., 2006) were also intensely stained by the CO histochemistry. Visual areas occupied the end-third of the isocortex and the auditory area appeared as a highly reactive oval-shaped region located in more lateral and posterior regions of the cortex, immediately caudal to S2 (Figure 2).

### Ipsilateral Connection Patterns Revealed by BDA

**FIGURE 7:**
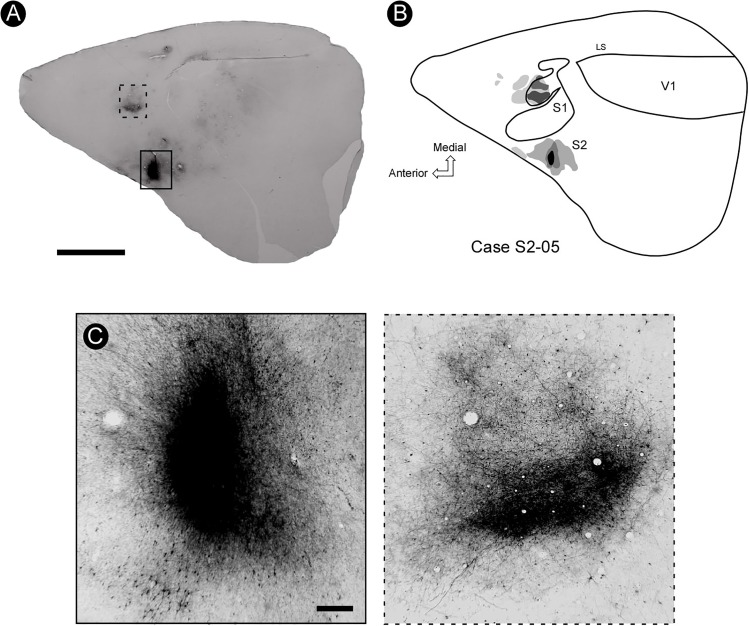
The pattern of connectivity between S2 and S1. **(A)** BDA injection (marked with a solid rectangle) in the representation of the forelimb in S2 is connected with its counterpart region in S1, as revealed by a dense focus of projection (dashed rectangle). **(B)** General reconstruction of a tangential section showing the site of injection of BDA in S2 and its projection to S1. It is possible to identify two foci located rostrally to this region, as well as a small focus near the rhinal fissure, probably area PR. **(C)** Enlargement of the site of injection in S2 (left) and the focus of projection in S1 (right). Scale bars: **A**: 5 mm; **B,C**: 500 μm. LS, lateral sulcus.

Injection of BDA in the forelimb representation of S2 resulted in a consistent pattern of intrinsic labeling defined by a central, strongly stained circular region surrounded by a less dense cloud where cell bodies, dendrites, and well-defined axon fragments could be discerned (Figure 7). The average diameter of the core injection region varied between 0.5 and 2.5 mm. A prominent homotopic ipsilateral connection from the forelimb representation in S2 to its counterpart in S1 was also found (Figures 7A,C), supporting the notion that similar body representation fields in S2 and S1 are strongly interconnected. Some heterotopic projections from the forelimb representation in S2 to the vibrissae representation in S1 were also identified (Figure 7B).

**FIGURE 8:**
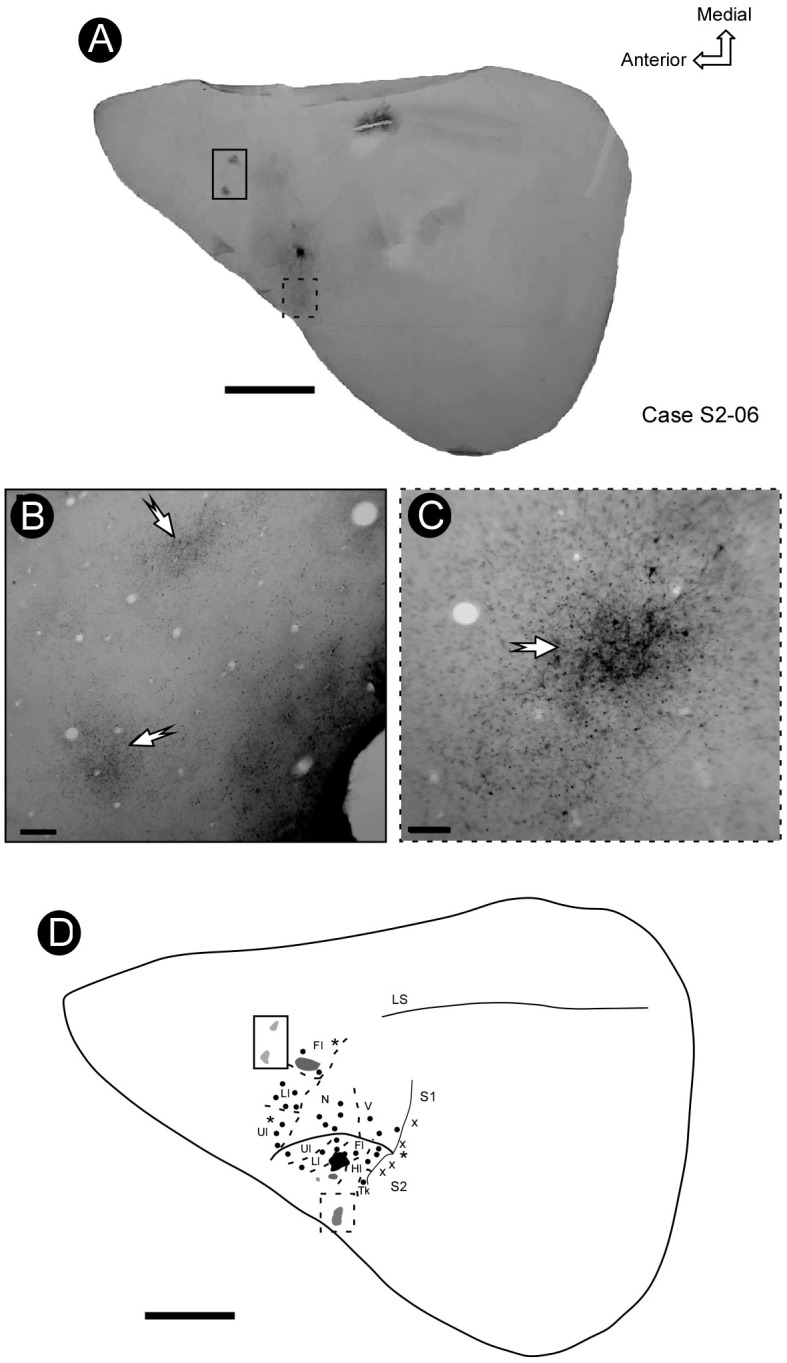
The pattern of connectivity between S2 and S1. **(A)** BDA injection in the representation of the forelimb in S2 (arrow). **(B,C)** Micrograph amplification showing the foci of projection from the region of forelimb representation in S2 to the rostral part of S1 (solid rectangle) and lateral part of S2 (dashed rectangle), respectively (arrows). **(D)** Electrophysiological mapping of S1 and S2, focusing on the representation of the forelimb, showing the site of injection in S2 (black spot), its projection to S1 (gray spot) and some additional foci located in a rostral region of S1 and area PR. Scale bars: **A,D**: 5 mm; **B,C**: 500 μm. LS, lateral sulcus.

In some cases, it was possible to identify a sparse focus of labeled projection to a region immediately rostral to S1, presumably corresponding to the motor cortex (Figures 8A,B). In addition, a less dense focus was also found in the more lateral and caudal regions of deeper tangential sections of the parietal cortex, close to the rhinal sulcus (Figure 8C), probably corresponding to area PR.

## Discussion

In the present work we describe the somatotopic organization and areal limits of the second somatosensory area (S2) located in the parietal cortex of a South American rodent, the agouti. We also compared the somatotopic map of the agouti’s S2 with both the pattern of intrahemispheric corticocortical connections originating in S2 and the chemoarchitectonic pattern revealed by CO histochemistry. Below, we discuss these findings in more detail.

### The Topographic Organization of Agouti’s S2: A Shared Rodent Plan

In addition to S1, there is at least one more lateral somatosensory area in mammals, corresponding to S2 (Kaas, 2000; Krubitzer and Kahn, 2003). This shared primitive trait is a synapomorphy retained from a common mammalian ancestor (see Northcutt and Kaas, 1995). Our present results show that, as in other mammals, the agouti’s S2 has a topographically organized representation of the contralateral body’s surface, with the representation of the face located more medially in the parietal cortex and the representations of both the forelimb and the hindlimb found more laterally, bordering the rhinal sulcus (Welker and Sinha, 1972; Campos and Welker, 1976; Nelson et al., 1979; Carvell and Simons, 1986; Krubitzer et al., 1986; Koralek et al., 1990; Slutsky et al., 2000; Remple et al., 2003; Sarko et al., 2011). A previous study by Pimentel-Souza et al. (1980) reported the presence of a bilateral representation of the body surface in the agouti’s S2. However, those authors used surface macro electrodes to map S2’s topography based on low-resolution slow-wave recordings, which may have contributed to the discordant results.

One important finding is that the basic organizational layout of cortical somatosensory fields in rodents, concerning both the number and organization of related areas, is retained in the lineage, independent of the volume of the brain (Divac, 1995; Clark et al., 2001). Thus, at least in the somatosensory cortex, the size of individual brain areas scales with overall brain volume. In all rodents studied so far S1 is densely and topographically connected with S2, even in rodents with markedly distinct lifestyles and habits such as the mouse, the squirrel and the fossorial naked-mole rat (Krubitzer et al., 1986; Carvell and Simons, 1987; Henry and Catania, 2006). Reciprocal corticocortical connections between homotypical regions of S1 and S2 areas have been systematically established in several mammalian lineages (Catania and Kaas, 2001; Disbrow et al., 2003; Liao and Yen, 2008). By the same token, we identified a similar correspondence in connectivity between S1 and S2 in the agouti, suggesting that this pattern remains across mammalian species with distinct habits, lifestyles and brain structure.

### Supplementary Somatosensory Areas in the Parietal Cortex

The matter of the presence and location of supplementary somatosensory areas in the parietal cortex of rodents remains undefined. Although PV has already been identified in the laboratory rat (Fabri and Burton, 1991; Remple et al., 2003), squirrels, marsupials and primates (see Karlen and Krubitzer, 2007; Kaas et al., 2018 for reviews) it has not been reported in the mouse (Carvell and Simons, 1986) and in some species of insectivores (Catania, 2000) and small marsupials (Huffman et al., 1999; Catania et al., 2000b). Even though we could not find an additional representation of the contralateral body periphery situated in the more lateral portion of the parietal cortex, we cannot rule out the existence of additional unimodal secondary somatosensory areas in the agouti, given that PV, for instance, may be located far more laterally in the parietal operculum and thus more inaccessible to electrophysiological recordings with conventional microelectrodes. Conversely, as seen in other rodents (Krubitzer et al., 1986; Brett-Green et al., 2004), it can be speculated that there are no additional secondary somatosensory areas besides S2 in this species. Though the agouti has a brain considerably larger than the rat’s, the smaller number of somatosensory cortical areas in the former may be associated with adaptations to a diurnal lifestyle (Divac, 1995), as seen in diurnal caviomorphs such as guinea pig and degu (Grant et al., 2017; Refinetti and Kenagy, 2018), and also in nocturnal non-caviomorphs (Campi and Krubitzer, 2010). Similar to another diurnal rodent, the squirrel, the agouti has a larger proportion of its dorsolateral cortex devoted to visual areas when compared to nocturnal rodents such as the rat (Picanço-Diniz et al., 1989; Campi and Krubitzer, 2010). On the other extreme, fossorial species such as the blind-mole-rat and the naked-mole-rat present a dramatic expansion of somatosensory areas that extend far into the occipital cortex (Necker et al., 1992; Henry et al., 2006), reinforcing the notion of changes in cortical organization in function of a species’ habits and lifestyle (Campi and Krubitzer, 2010).

### Connections From S2 in Agouti

Evidence from other mammalian species support the notion that S2 is very well interconnected ipsilaterally with other somatosensory cortical fields [primates (Burton and Carlson, 1986; Weller and Kaas, 1987; Krubitzer and Kaas, 1990; Disbrow et al., 2003), carnivores (Hartenstein et al., 1980; Herron and Johnson, 1987), insectivores (Catania and Kaas, 2001), rodents (Krubitzer et al., 1986; Carvell and Simons, 1987; Koralek et al., 1990), marsupials (Beck et al., 1996; Elston and Manger, 1999)]. In the present study, we found a small focus of projection to deep layers of a lateral region of the parietal cortex, next to the rhinal sulcus, not reported in a previous work with the agouti (Pimentel-Souza et al., 1980). Based in its relative location, we propose that this area corresponds to the multimodal (auditory, somatosensory) parietal rhinal area (PR), first reported by Krubitzer et al. (1986) in squirrels and subsequently described in other species (Krubitzer and Kaas, 1990; Fabri and Burton, 1991).

S2 is also interconnected with both S1 and S2 of the contralateral hemisphere (Krubitzer and Kaas, 1990; Guillemot et al., 1992; Hayama and Ogawa, 1997; Catania and Kaas, 2001). The distribution of callosal connections in the mammalian somatosensory cortex is frequently interpreted in the perspective of the “midline fusion hypothesis,” which holds that one critical function of callosal connections is to generate a contiguous sensory map by connecting the representations of midline body parts in each hemisphere (Guillemot et al., 1992). Our previous results in the agouti’s somatosensory cortex are in agreement with the midline hypothesis since more lateral regions of the body representation in S1 and S2 were homotopically connected with their counterparts in the contralateral hemisphere (Rocha et al., 2007); in that study we showed that callosal projections were restricted to sites in S1 and S2 in the contralateral hemisphere. Though not the focus of the present work, the absence of labeled terminals in other contralateral areas can be explained in part by the relatively small amount of neurotracer we used, in a pattern that replicates the findings of other studies (Catania and Kaas, 1997, 2001).

### Histochemical Limits of the Agouti’s Sensory Areas

The pattern of CO reactivity in tangential sections revealed that the organization of the agouti’s auditory, somatosensory, and visual areas is similar to those described previously in the agouti and in small rodents by both histological and histochemical techniques (Wallace, 1987; Elston et al., 2006; Rocha et al., 2007, 2012; Freire et al., 2010, 2012). In addition, CO revealed the presence of conspicuous modules in the region of the face representation of S1, as previously described in other rodents and marsupials (Woolsey et al., 1975; Wallace, 1987; Weller, 1993; Pereira et al., 2000; Freire et al., 2005; Sarko et al., 2011). However, similar to other rodents, we did not identify the presence of cortical modules in S2 (Remple et al., 2003; Krubitzer et al., 2011; Sarko et al., 2011).

## Conclusion

**FIGURE 9:**
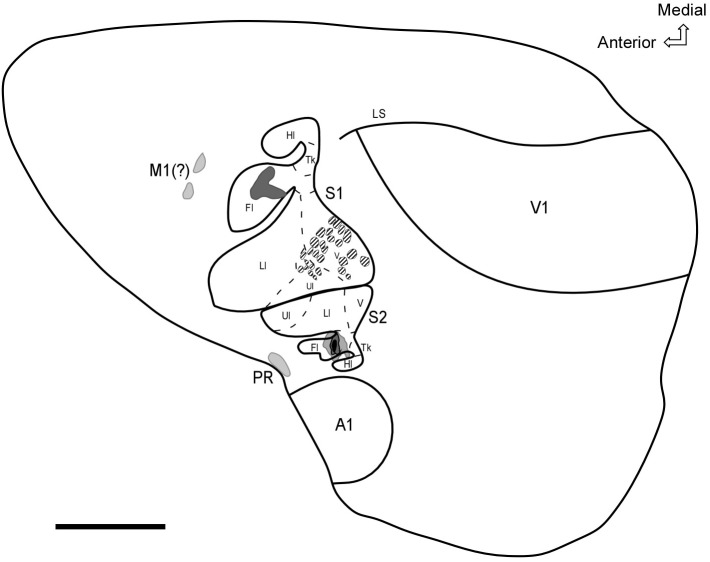
Schematic diagram representing the general organization of sensory areas and feed forward projections from the forelimb representation in S2. The shaded areas represent projection targets in S1, the motor cortex, and PR. CO modules located in the face area of S1 are represented by hatched areas. Scale bar: 3 mm. Fl, forelimb; Hl, hindlimb; Ll, lower lip; N, nose; Tk, trunk; Ul, upper lip; V, vibrissae; A1, Primary auditory cortex; V1, primary visual cortex; LS, lateral sulcus.

In summary, our results revealed the existence of a secondary somatosensory field (S2) with a mirror-reversed representation of S1 and located more laterally in the parietal cortex of the agouti. In addition, neurotracer injections into the forelimb representation of S2 revealed a dense homotopic ipsilateral projection to S1, supplemented by a less dense projection to PR located in the caudolateral cortex close to the rhinal sulcus and also to a frontal region probably associated with the motor cortex (see Figure 9).

The relative location, somatotopic organization and interconnections of the agouti’s S2 are similar to those described in other species, suggesting a common plan of organization for the second somatosensory area in the mammalian parietal cortex. The absence of a third somatosensory area in the agouti, such as PV, may represent a distinct feature of this diurnal species in relation to other rodents with nocturnal habits. The allocation of cortical tissue to the processing of sensory information depends on a species lifestyle, as seen with the presence of barrels in S1 or the expansion of cortical areas devoted to visual processing in diurnal rodent species.

## Ethics Statement

This study was carried out in accordance with the recommendations of the Ethics Committee for the Experimental Use of Animals of the Federal University of Pará. The protocol was approved by the Ethics Committee for the Experimental Use of Animals of the Federal University of Pará under license from the Brazilian Institute of the Environment and Renewable Natural Resources (IBAMA) – Brazil.

## Author Contributions

AP, JF, MF, and LS designed the study and acquired the data. AP, JF, and CP-D contributed to reagents, materials, and analysis tools. All authors analyzed and interpreted the data and wrote the manuscript.

## Conflict of Interest Statement

The authors declare that the research was conducted in the absence of any commercial or financial relationships that could be construed as a potential conflict of interest.

## Footnotes

1 https://imagej.nih.gov/ij/
